# Network Pharmacology and Bioinformatics Approach Reveals the Therapeutic Mechanism of Action of Baicalein in Hepatocellular Carcinoma

**DOI:** 10.1155/2019/7518374

**Published:** 2019-02-12

**Authors:** Chongyang Ma, Tian Xu, Xiaoguang Sun, Shuang Zhang, Shuling Liu, Shuning Fan, Chaofang Lei, Feifei Tang, Changming Zhai, Changxiang Li, Juan Luo, Qingguo Wang, Wei Wei, Xueqian Wang, Fafeng Cheng

**Affiliations:** ^1^School of Traditional Chinese Medicine, Beijing University of Chinese Medicine, Beijing 100029, China; ^2^Department of Gastroenterology, Wangjing Hospital of China Academy of Chinese Medical Sciences, Beijing 100102, China

## Abstract

Liver cancer is the fourth leading cause of cancer death worldwide, and hepatocellular carcinoma (HCC) accounts for the greatest proportion of these deaths. Baicalein, a flavonoid isolated from the root of* Scutellariae radix*, is considered a potential candidate to treat HCC. However, the underlying molecular mechanisms remain poorly understood. In the present study, a network pharmacological approach was combined with microarray data (GSE95504) acquired from the Gene Expression Omnibus database to reveal the therapeutic mechanisms of action of baicalein at a systemic level. We identified 38 baicalein targets and 76 differently expressed genes (DEGs) following treatment with baicalein, including 55 upregulated and 21 downregulated genes. The DEGs were significantly enriched in the biological functions of apoptosis, endoplasmic reticulum stress, and PERK-mediated unfolded protein response. Protein-protein interaction (PPI) network construction and topological screening revealed a core module of PPIs including two baicalein targets,* TP53* and* CDK1*, and two downregulated DEGs,* HSPA1A* and* HSPA1B*. Expression and survival data for these genes in the module derived from Gene Expression Profiling Interactive Analysis (GEPIA) were subjected to Kaplan–Meier analysis of overall survival and disease-free survival. Overexpression of* CDK1*,* BRCA1*,* TUBB*,* HSPA1A*,* HSPA1B*, and* HSPA4* was associated with significantly worse overall survival, while overexpression of* CDK1*,* CLU7*,* BRCA1*, and* TUBB* was associated with significantly worse disease-free survival. These data suggest that baicalein exerts therapeutic effects against HCC via a PPI network involving TP53, CDK1, HSPA1A, and HSPA1B.

## 1. Introduction

Liver cancer is the fourth leading cause of cancer death worldwide according to global cancer statistics for 2018, with about 782,000 deaths annually [1]. Hepatocellular carcinoma (HCC), comprising 75%–85% of cases, is the most common type of primary liver cancer and is strongly associated with chronic infection with hepatitis B virus (HBV) or hepatitis C virus (HCV), consumption of aflatoxin-contaminated foodstuffs, and heavy alcohol intake [2]. Although improvements in patient outcomes were observed in clinical trials with first- or second-line drugs, the lack of improvements in overall survival in some patients and the lack of curative treatment options must be urgently addressed [3]. Therefore, elucidation of the molecular mechanisms underlying HCC and development of alternative therapies with lower toxicity is critical for achieving more favorable clinical outcomes and reducing treatment morbidity.

The use of traditional Chinese medicine to treat cancer is built on a foundation of more than 2,500 years of Chinese medical practice, including Chinese herbal medicine (CHM), acupuncture, and dietary therapy. A recently published meta-analysis of 20 randomized controlled trials showed that add-on therapy with CHM improved overall survival in HCC patients and reduced adverse events related to conventional treatments [4]. To date, ingredients derived from CHM have been found to exert suppressive effects on the promotion and proliferation of cancer cells, as well as inhibiting angiogenesis in cancer tissues [5]. Baicalein, a compound originally isolated from* Scutellariae radix*, is reported to exert anticancer activity by blocking colony formation, activating apoptosis, inducing autophagy, and modulating molecular signaling pathways such as mTOR and Wnt/*β*-catenin in several human liver cancer cell lines [6–8]. Multitarget therapies elicit complex changes in multiple biological networks and mechanisms and are therefore considered an effective approach for treating HCC [9].

In contrast to the traditional “one drug, one target” principle of drug design, network pharmacology aims to investigate the influence or intervention of drugs on diseases as a whole, based on the synergism of multitargeted drugs and through a holistic perspective at a systemic level [10]. This approach encompasses systems biology, network analysis, connectivity, redundancy, and pleiotropy [11]. Recently, a network pharmacological platform was successfully applied for screening effective ingredients and revealing the pharmacological mechanisms of CHM. Whether by studying classic formulae, patented modern Chinese medicines, or bioactive ingredients derived from CHM, the network pharmacological approach provides new insights into the systemic connection between CHM, therapeutic targets, and a disease as a whole and provides a powerful and promising tool for the elucidation of disease mechanisms at a systemic level and the discovery of potential bioactive ingredients [12–14].

In the present study, in order to recognize the mechanisms underlying the anti-HCC effects of baicalein, we used a network pharmacological approach, including the evaluation of its absorption, distribution, metabolism, and excretion (ADME) properties; identification of differentially expressed genes (DEGs) related to baicalein treatment; prediction of baicalein-related targets; and recognition of core functions and modules via the protein-protein interaction (PPI) network approach. We identified a core modulatory network including two important baicalein targets, TP53 and CDK1 that was significantly related to the regulation of cell death and modulation of the apoptotic signaling pathway. To our knowledge, this study is the first to use a network pharmacological approach to study the efficacy and mechanism of baicalein, providing a powerful and promising tool for the development and application of CHM to HCC therapy.

## 2. Materials and Methods

### 2.1. Public Data Collection

The Gene Expression Omnibus (GEO, http://www.ncbi.nlm.nih.gov/geo) is a public functional genomics data repository of high-throughput gene expression, chip, and microarray data. We downloaded the data series GSE95504 from the GEO database (species:* Homo sapiens*), which contains samples of the HCC cell line Bel-7402 treated with DMSO and 40 *μ*M baicalein. All samples were processed on the GPL17586 Affymetrix Human Transcriptome Array 2.0 platform. The probes were represented using the corresponding gene symbol according to the annotation information in the platform.

### 2.2. Identification of DEGs

The microarray data were preprocessed using RMA with the oligo and limma package in Bioconductor (v3.7; http://www.bioconductor.org/) [15]. Background correction, normalization, and calculation of expression were all included in the preprocessing stage. Microarray data probe IDs were transformed into gene symbols with the Bioconductor Annotation Data software packages. When several probes mapped to the same gene symbol, the mean value was set as the final expression value of this gene. P values were acquired using the unpaired Student's* t*-test provided by the limma package and were adjusted using the Benjamin–Hochberg (BH) method. The thresholds for identifying DEGs were set as adjusted (adj.) P < 0.05 and |log_2_ fold change (FC)| > 1. Hierarchical clustering analysis of the DEGs was then performed and visualized using g-plots as implemented in the R package.

### 2.3. Evaluation of Drug-Likeness (DL)

The TCMSP server (http://lsp.nwu.edu.cn/tcmsp.php) is a systems-level pharmacological database for traditional Chinese medicine that can also be used for calculating the ADME-related properties of naturally occurring compounds of interest [16]. It provides an* in silico* ADME-systems evaluation model created by Wang et al., which integrates DL, oral bioavailability (OB), Caco-2 permeability, and other features.

### 2.4. Prediction of Baicalein Targets

We used the drug targeting* in silico* prediction models developed by Wang and others to identify potential targets for baicalein [17]. In brief, the* in silico* prediction model efficiently integrates chemical, genomic, and pharmacological information for drug targeting on a large scale, based on two powerful methods: random forest (RF) and support vector machines (SVM). In cases in which drug targets are identified, proteins with an output expectation value (E-value) for SVM > 0.7 or RF > 0.8 are listed as potential targets.

### 2.5. PPI Network Construction

We used BisoGenet [18], a Cytoscape plugin, to construct a PPI network using six currently available PPI databases, including the Biological General Repository for Interaction Datasets (BioGRID), Biomolecular Interaction Network Database (BIND), Molecular INTeraction Database (MINT), Human Protein Reference Database (HPRD), and Database of Interacting Proteins (DIP). After interactive networks for putative baicalein targets and DEGs were constructed using Cytoscape [19], a merged network was constructed based on the intersection data of the two networks.

### 2.6. Definition of Topological Feature Set for the Network

We used CytoNCA [20], a Cytoscape plugin, to analyze the topological properties of every node in the interaction network in order to calculate two topological properties: betweenness centrality (BC) and degree centrality (DC). More important nodes received higher quantitative values within the network.

### 2.7. Clusters of Core PPI Networks

We used MCODE, a plugin of Cytoscape, to obtain clusters of core PPI networks by analyzing the corresponding networks [21, 22]. Based on network theory, connected regions in large PPI networks may represent molecular complexes and together disrupt biological functions, resulting in a particular disease phenotype. As the topological module and functional module have the same meaning in the network, the functional module can be recognized by network properties.

### 2.8. Gene Expression Data for the Core Cluster for HCC

Data were obtained from the Gene Expression Profiling Interactive Analysis (GEPIA) online database (http://gepia.cancer-pku.cn/), a web server that provides customizable functions [23]. Tumors and normal samples in the GEPIA database were derived from The Cancer Genome Atlas (TCGA) and the Genotype-Tissue Expression (GTEx) projects. Correlations of disease-free survival and overall survival rates with the expression levels of* CDK1*,* CUL7*,* BRCA1*,* TUBB*,* HSPA1A*,* HSPA1B*, and* HSPA4* in HCC patients were also computed using the GEPIA database.

### 2.9. Gene Ontology and Pathway Enrichment Analysis

We used the Database for Annotation, Visualization, and Integrated Discovery (DAVID; http://david.abcc.ncifcrf.gov/) [24], an online program that provides comprehensive data for high-throughput gene functional analysis for elucidation of biological characteristics, to obtain Gene Ontology (GO) terms belonging to the biological process (BP), cellular component (CC), and molecular function (MF) categories. Additionally, Kyoto Encyclopedia of Genes and Genomes (KEGG) pathway functional enrichment analyses were performed for the above DEGs. Results with values of P < 0.05 were considered to be statistically significant. We performed KEGG signaling pathway enrichment analysis of the screened candidate targets of baicalein using ClueGO, a Cytoscape plugin, to visualize nonredundant biological terms for large clusters of genes in a functionally grouped network [25]. The ClueGO network was created with kappa statistics and reflects the relationship between the terms based on the similarity of their associated genes.

## 3. Results

### 3.1. Identification of DEGs

**Figure 1:**
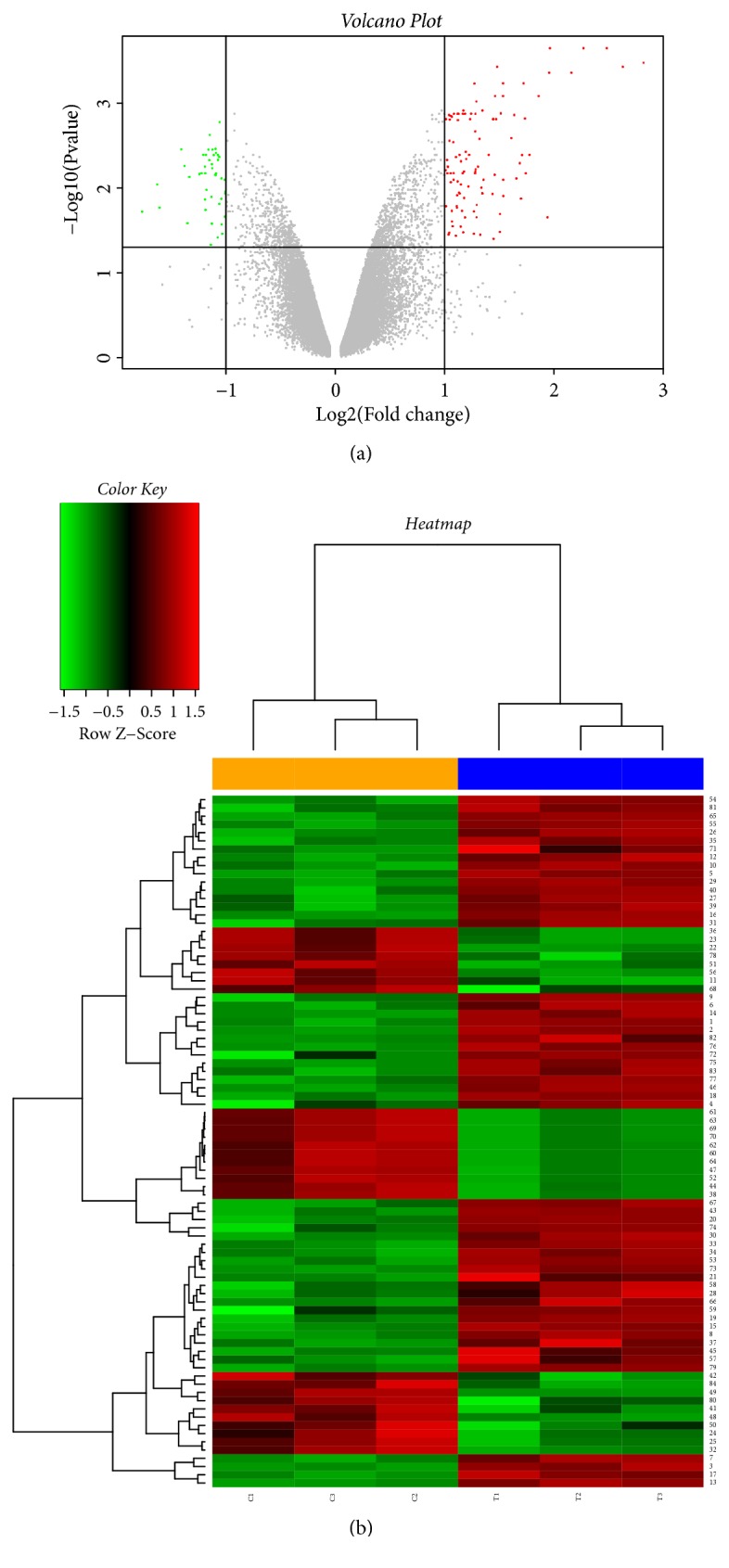
Volcano plot of gene expression and heatmap of DEGs from GSE95504. (a) The x-axis specifies the log_2_ fold change (FC), and the y-axis specifies the –log_10_ of the adj. P-value. Black vertical and horizontal lines reflect the filtering criteria (adj. P < 0.05 and |log_2_⁡FC| > 1). (b) Rows represent genes, and columns represent samples. The heatmap is color-coded based on the Z-score; red represents a high expression value and green represents a low expression value.

Gene expression dataset GSE95504 was downloaded from the GEO database. Statistical analysis software R was used for preprocessing and gene differential expression analysis of microarray data. A total of 76 DEGs were identified between HCC BEL-7402 cells treated with and without baicalein, including 55 that were upregulated and 21 that were downregulated upon baicalein treatment, for subsequent bioinformatics analysis (Table 1, Figure 1).

### 3.2. GO Enrichment Analyses of DEGs

**Figure 2:**
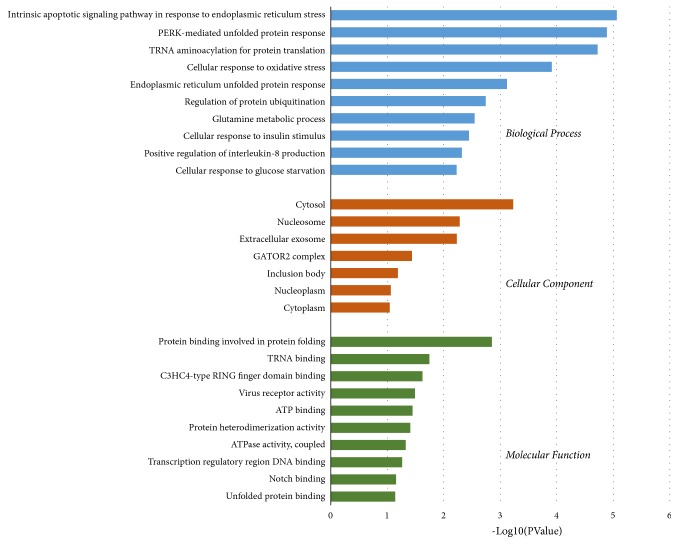
GO analysis for biological process, cellular component, and molecular function terms was performed on DEGs; the top 10 terms with P < 0.05 are shown.

To analyze the biological classifications of DEGs, functional enrichment analyses were performed using the DAVID server. As shown in Figure 2, GO analysis results indicated that, in the BP category, DEGs were significantly enriched in the intrinsic apoptotic signaling pathway in response to endoplasmic reticulum stress, PERK-mediated unfolded protein response, tRNA aminoacylation for protein translation, cellular response to oxidative stress, endoplasmic reticulum unfolded protein response, regulation of protein ubiquitination, glutamine metabolic process, cellular response to insulin stimulus, positive regulation of interleukin-8 production, and cellular response to glucose starvation. In the CC category, DEGs were mostly enriched in the cytosol, nucleosome, extracellular exosome, GATOR2 complex, inclusion body, nucleoplasm, and cytoplasm. In the MF category, DEGs were mainly enriched in protein binding involved in protein folding, tRNA binding, C3HC4-type RING finger domain binding, virus receptor activity, ATP binding, protein heterodimerization activity, ATPase activity, transcription regulatory region DNA binding, Notch binding, and unfolded protein binding.

### 3.3. Baicalein ADME Properties, Target Prediction, and PPI Network Construction

**Figure 3:**
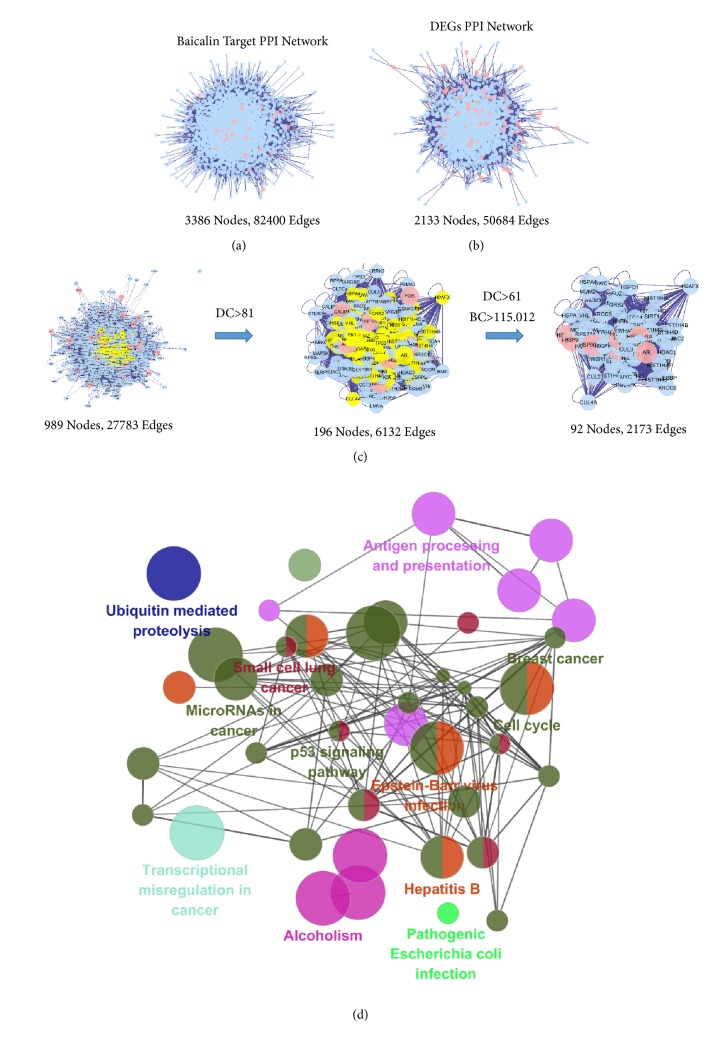
Identification and KEGG enrichment analysis of candidate targets for baicalein against HCC. (a) Protein-protein interaction network of baicalein targets. (b) Protein-protein interaction network of DEGs. (c) Topological screening of the interactive PPI network of baicalein targets and DEGs based on degree and betweenness centrality. (d) KEGG enrichment analysis was performed by ClueGO, and the most vital term in the group is labeled.

As shown in Table 2, the DL value of baicalin was 0.21, OB value of baicalin was 33.52%, and Caco-2 permeability of baicalin was 0.63, indicating the potential applicability of baicalin as an oral drug agent. We further predicted 38 potential targets of baicalein including TP53 and CDK1 (Table 3). PPI networks were constructed of baicalein targets and DEGs. We further merged the intersection of the two networks and obtained a core network (92 nodes, 2173 edges) according to the DC and BC (Figures 3(a)–3(c)).

### 3.4. KEGG Signaling Pathway Enrichment and Main Modules of Core PPI Network Recognition

**Figure 4:**
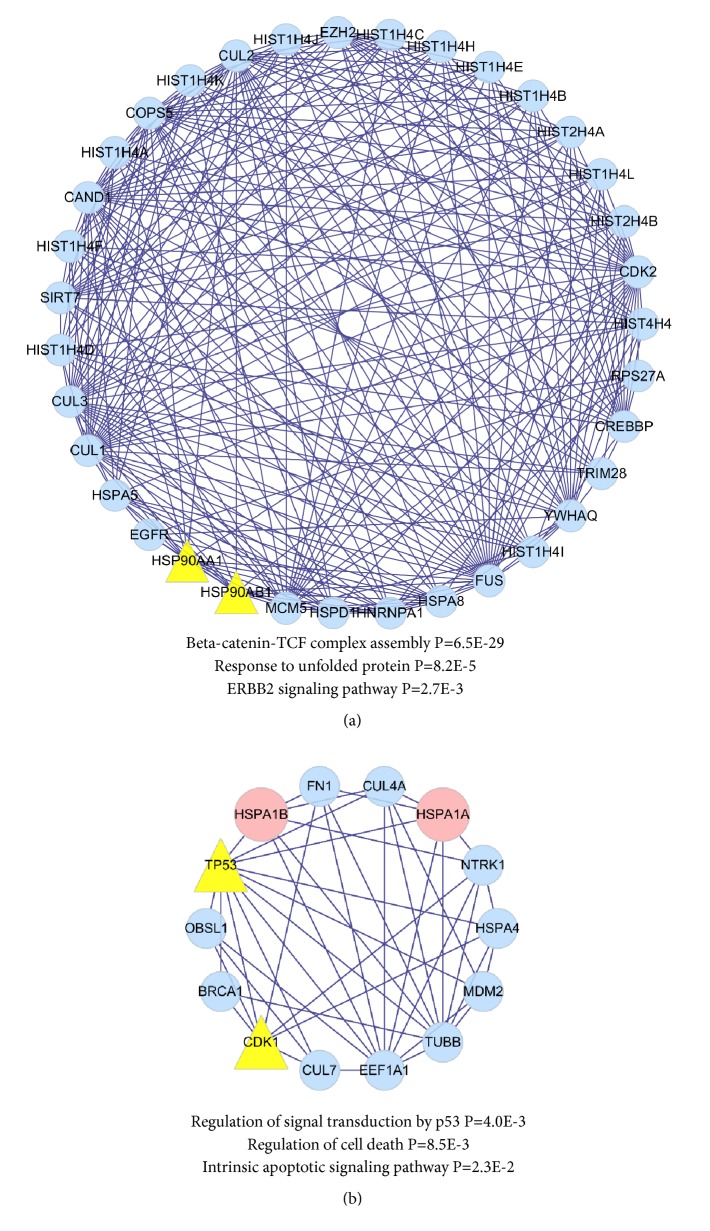
Modules of core PPI networks of baicalein against HCC. (a) One module was related to beta-catenin-TCF complex assembly, response to unfolded protein, and ERBB2 signaling pathway. (b) The other module was related to regulation of signal transduction by p53, regulation of cell death, and intrinsic apoptotic signaling pathway.

KEGG signaling pathway enrichment using ClueGO indicated that proteins in the core PPI network were statistically enriched for the following pathways: Epstein-Barr virus infection, bladder cancer, antigen processing and presentation, cell cycle, alcoholism, ubiquitin-mediated proteolysis, and transcriptional misregulation in cancer. Other enriched pathways included the p53 signaling pathway, breast cancer, colorectal cancer, hepatitis B, and microRNAs in cancer (Figure 3(d)). A Cytoscape plugin, MCODE, was used to carry out module analysis, resulting in two main modules of the core PPI network (Figure 4). The first module, containing two baicalein targets, was functionally enriched in beta-catenin-TCF complex assembly, response to unfolded protein, and the ERBB2 signaling pathway. The second module, containing two baicalein targets and two DEGs, was enriched in regulation of signal transduction by p53, regulation of cell death, and the intrinsic apoptotic signaling pathway.

### 3.5. Analysis of HCC Survival and Expression of Proteins in the Module of Interest

**Figure 5:**
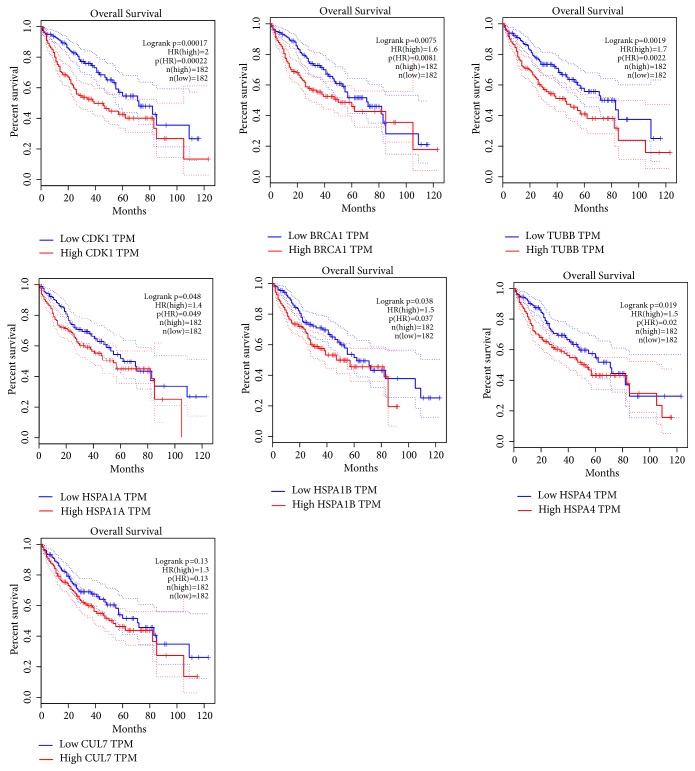
Overall survival analyses of* CDK1*,* CUL7*,* BRCA1*,* TUBB*,* HSPA1A*,* HSPA1B*, and* HSPA4* were performed using GEPIA online platform; differences with P < 0.05 were considered statistically significant.

**Figure 6:**
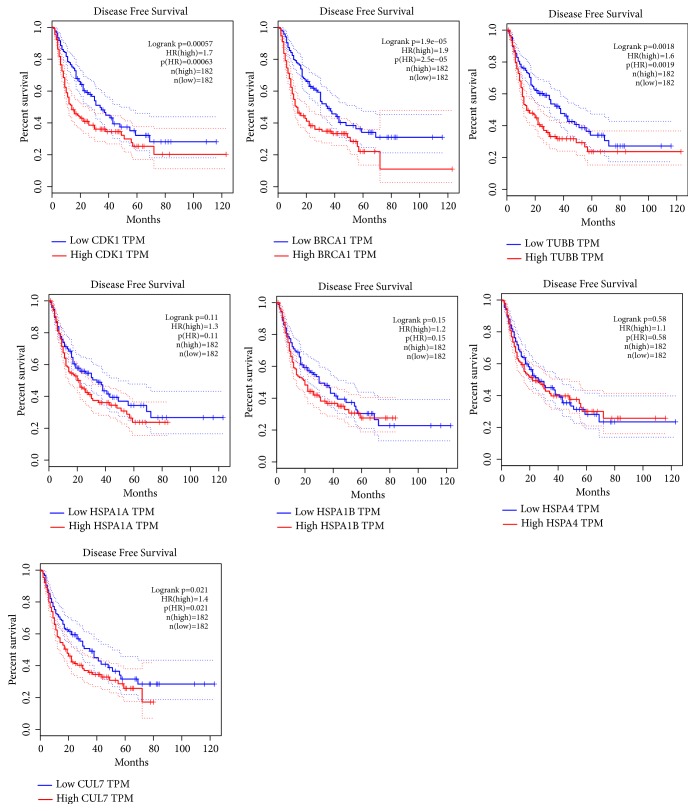
Disease-free survival analyses of* CDK1*,* CUL7*,* BRCA1*,* TUBB*,* HSPA1A*,* HSPA1B*, and* HSPA4* were performed using GEPIA online platform; differences with P < 0.05 were considered statistically significant.

We used the GEPIA online database to perform overall survival and disease-free survival analyses for proteins in module 2 using a Kaplan–Meier curve. HCC patients with higher expression of* CDK1*,* BRCA1*,* TUBB*,* HSPA1A*,* HSPA1B*, and* HSPA4* showed significantly worse overall survival (Figure 5), while HCC patients with higher expression of* CDK1*,* CLU7*,* BRCA1*, and* TUBB* showed significantly worse disease-free survival (Figure 6).

## 4. Discussion

*S. baicalensis* is a skullcap plant native to several East Asian countries and Russia, and it is cultivated in many European countries. This plant has been widely used in traditional Chinese medicine since about 200–250 AD for the clinical treatment of hypertension, atherosclerosis, dysentery, common colds, inflammatory disorders, and tumors [26–28]. Baicalein is a major bioactive flavone derived from the dry roots of* S. baicalensis*. It has been shown that treatment with baicalein inhibits cell proliferation and enhances apoptosis and autophagy of many types of cancer, including HCC [29], bladder cancer [30], and colorectal cancer [31]. Previously, studies have also demonstrated that baicalein exerts multiple effects against HCC, mainly involving the inhibition of cell proliferation by arresting the cell cycle, suppression of metastasis, induction of apoptosis, as well as induction of autophagy via the modulation of associated molecules and signaling pathways [32]. However, these studies were designed based on the traditional research idea of “one drug, one target”, without considering a systemic picture. Therefore, in the present study, we used a network pharmacological strategy to explore the molecular mechanisms by which baicalein exerts therapeutic effects on HCC from a systemic perspective, and we sought to establish connections between basic and clinical research.

We used* in silico* approaches to determine whether baicalein is a good candidate for drug discovery for HCC. According to Lipinski's “rule of five”, which predicts the DL of a chemical compound with a certain biological activity designed for administration via the oral route [33], the properties of baicalein meet the requirements, namely, molecular weight < 500 Da, calculated log⁡P < 5, number of hydrogen-bond donors < 5, and number of hydrogen-bond acceptors < 10, indicating that this compound is a suitable potential candidate for drug discovery for HCC. Based on data from the TCMSP database, the DL of baicalein was calculated to be 0.21, which is above the average DL value of DrugBank compounds (0.18); these data additionally indicate the potential of baicalein as a promising candidate for HCC treatment.

To understand the anti-HCC effect of baicalein at a systemic level, we predicted targets of baicalein and analyzed microarray data from baicalein-treated HCC cell lines derived from the GEO database. Thirty-eight baicalein targets and 76 DEGs were identified and selected for further analysis. GO analysis of DEGs indicated that apoptosis, endoplasmic reticulum stress, and oxidative stress were statistically related to the anti-HCC effect of baicalein. Through PPI network construction and topological screening, we obtained a core PPI network with 92 nodes and 2173 edges. Following KEGG analysis of proteins from the core PPI network using ClueGO, we identified the Epstein-Barr virus infection, cell cycle, alcoholism, hepatitis B, and p53 signaling pathways, as well as some cancer pathways. An increasing number of studies support the idea that baicalein induces the apoptosis of HCC cells and that the underlying biological mechanisms are strongly related to endoplasmic reticulum stress, reactive oxygen species generation, and the p53 signaling pathway [32, 34, 35]. Baicalein has also been shown to arrest the cell cycle in different HCC cell lines at all three phases: G0/G1 [36], S [37], and G2/M [38]. These findings are consistent with the results from the present* in silico* network pharmacological analysis.

Using a topological approach, we identified an important module containing 14 genes, including* TP53* (baicalein target),* CDK1* (baicalein target),* HSPA1B* (downregulated DEG),* HSPA1B* (downregulated DEG), and other genes in the PPI network; these genes play important roles in the regulation of signal transduction by p53, regulation of cell death, and the intrinsic apoptotic signaling pathway. We further assessed the expression of these genes in relation to overall and disease-free survival. High expression of* CDK1*,* BRCA1*, and* TUBB* was significantly associated with reductions in both overall and disease-free survival.

Baicalein has been identified as a selective inhibitor of CDK1 by FRET analysis and structure-activity relationship analysis in the Hep G2 and SMMC-7721 cell lines [39, 40]. A549 and H1299 non-small-cell lung cancer cells and primary cultured heart endothelial cells treated with baicalein have additionally been reported to exhibit downregulated expression of CDK1 [41]. CDK1 is a member of the Ser/Thr protein kinase family that is frequently overexpressed in HCC and associated with tumor progression [42, 43]. Depletion or inhibition of CDK1 with microRNAs or small molecular compounds has been shown to lead to reduced clonogenicity by arresting cells in the S–G2 and G2–M phases of the cell cycle and inducing apoptosis in HCC cell lines [44–47]. TP53, another baicalein target, is a classic tumor suppressor involved in tumor cell apoptosis, genomic stability, and inhibition of angiogenesis [48]. Previous studies have found that baicalein induces cell cycle arrest and apoptosis in HepG2 cells via a TP53-dependent pathway [49]. Similar results were observed in human lung [50], colorectal [51], and prostate [52] cancer cells.

Heat shock proteins (HSPs) and molecular chaperones are considered to play important roles in protein homeostasis, cell physiology, and protection against stressors [53]. In the present study, HSPA1A and HSPA1B represented two downregulated DEGs present in the PPI module network of baicalein. As members of the heat shock protein 70 (Hsp70) family, they were similarly expressed in both tumor and nontumor tissues of HCC patients, with higher expression levels in HCC tumor tissues [54]. Previous studies have shown that the knockdown of HSP70 inhibits the proliferation of two HCC cell lines, namely SMMC-7721 and Hep3B cells [55].* In vitro* evidence showed that high levels of HSP70 promote cell migratory ability and suppress apoptosis [56]. Of note, it has been reported that HSP70 plays significant roles in endothelial cell migration and lumen formation and is considered an important molecule in angiogenesis and the immune response in HCC [57].

To further understand the roles of the ten other proteins in the potential module, based on our current knowledge, we propose that EEF1A1, MDM2, CUL7, and BRCA1 are strongly related to HCC. These proteins are considered to play important roles in the pathophysiology of tumors and participate in cell growth, cell cycle regulation, and the maintenance of cell survival [58–61].

In conclusion, our* in silico* study showed that the anti-HCC effect of baicalein was related to endoplasmic reticulum stress, apoptosis, oxidative stress, and the p53 signaling pathway. We identified a PPI network module containing 14 proteins, and we speculate that baicalein targets CDK1 and TP53 to downregulate the expression of HSP70, thereby exerting preventive effects against HCC via this module. Biological experiments are needed to verify these* in silico* results in the future.

## Figures and Tables

**Table 1:** Differently expressed genes from GSE95504.

Gene	log⁡FC	AveExpr	t	P-Value	adj. P-Value	B
FAM129A	2.624683	7.781198	21.34116	3.19E-08	0.000375	8.410651
ASNS	2.267513	7.745739	25.81981	7.31E-09	0.000226	9.168015
DDIT3	2.158061	5.129843	20.13082	5.00E-08	0.000441	8.148515
SLC7A11	1.940169	8.499572	6.547421	0.000196	0.022113	1.219042
CTH	1.859453	6.75212	17.32877	1.58E-07	0.000828	7.413078
SLFN5	1.77625	7.65223	11.24138	4.16E-06	0.004075	4.860584
ULBP1	1.734087	5.290091	14.11018	7.56E-07	0.001523	6.270654
OSBPL6	1.721142	5.858125	18.94741	7.97E-08	0.000586	7.861891
PSAT1P4	1.708273	7.674072	11.01701	4.83E-06	0.004101	4.729785
CLDN1	1.63562	6.888961	14.4617	6.27E-07	0.001383	6.414871
GDF15	1.610927	6.988729	12.48127	1.90E-06	0.002583	5.525689
CLGN	1.536074	4.371125	18.84393	8.31E-08	0.000586	7.835261
TRIB3	1.535661	7.709365	17.24218	1.64E-07	0.000828	7.386996
TUBE1	1.515188	5.96114	15.58245	3.56E-07	0.00132	6.840486
ERRFI1	1.482139	7.451778	21.4253	3.09E-08	0.000375	8.427814
GPR1	1.471302	4.486647	13.82075	8.84E-07	0.001541	6.147765
GFPT1	1.462896	8.845707	17.25109	1.64E-07	0.000828	7.389692
PXK	1.447934	5.986157	13.87282	8.59E-07	0.001541	6.170157
PSAT1	1.443356	10.30946	13.75243	9.18E-07	0.001541	6.118191
NFIL3	1.438356	6.135237	7.852097	5.60E-05	0.011763	2.454479
PMAIP1	1.319246	6.572005	12.40886	1.99E-06	0.002647	5.489364
CHAC1	1.30625	7.242645	9.979721	1.00E-05	0.005625	4.078163
C15orf65	1.290543	6.123697	6.54843	0.000196	0.022113	1.220081
GADD45A	1.289319	7.282376	16.76291	2.04E-07	0.000958	7.238494
WARS	1.275291	9.228478	12.87648	1.51E-06	0.002159	5.718873
SEL1L3	1.274108	7.258999	9.408758	1.54E-05	0.006705	3.683429
STC2	1.238835	6.081823	15.4665	3.77E-07	0.001328	6.798729
SLC22A15	1.22956	6.064722	13.9939	8.05E-07	0.001541	6.221742
CTAGE5	1.220954	6.554274	11.07457	4.64E-06	0.004075	4.763668
JAG1	1.214166	5.838658	8.328888	3.69E-05	0.009608	2.856702
SESN2	1.19303	7.048649	10.73854	5.83E-06	0.004571	4.562614
PHGDH	1.18876	7.266541	11.47118	3.58E-06	0.003764	4.991071
GARS	1.184448	9.965868	14.75109	5.40E-07	0.001333	6.529612
SGTB	1.173656	5.908581	7.658115	6.68E-05	0.013087	2.283709
MOCOS	1.16865	8.951834	15.92122	3.02E-07	0.001224	6.959663
PDE10A	1.122733	6.077276	14.89259	5.02E-07	0.001333	6.584453
RND3	1.118622	6.761436	13.61063	9.92E-07	0.001591	6.056112
PCK2	1.11477	6.688267	15.34161	4.01E-07	0.001333	6.753186
ARG2	1.109017	5.776241	7.100543	0.000113	0.016946	1.768427
CCDC113	1.10665	6.174742	7.187338	0.000104	0.016349	1.851102
ZNF252P	1.100149	6.009432	5.675607	0.000504	0.03647	0.26837
CARS	1.082632	6.759968	14.92228	4.94E-07	0.001333	6.595858
ETV5	1.080051	6.215242	9.459451	1.48E-05	0.00668	3.719593
AARS	1.075095	10.13323	12.06077	2.46E-06	0.002893	5.310541
NR1D2	1.069287	6.525232	6.13772	0.000302	0.02816	0.785742
PYROXD1	1.064665	7.7943	6.357469	0.000239	0.024807	1.021033
TRIM2	1.063894	6.068377	14.25706	6.99E-07	0.001449	6.331573
XPOT	1.052345	9.70088	8.58299	2.98E-05	0.008532	3.061361
HERPUD1	1.051263	8.736178	14.36397	6.60E-07	0.001411	6.375315
XBP1	1.043021	7.951268	13.68627	9.52E-07	0.001561	6.089348
SARS	1.039932	8.565683	12.23496	2.21E-06	0.002784	5.400901
ACAD11	1.036179	5.605899	14.46101	6.27E-07	0.001383	6.414591
TSEN15	1.027667	6.697341	9.904686	1.06E-05	0.005645	4.02783
BMP6	1.020646	8.133826	9.363714	1.59E-05	0.006705	3.651105
NUPR1	1.018416	8.725291	10.60041	6.42E-06	0.004666	4.477638
S1PR3	-1.00496	5.622551	-7.97262	5.03E-05	0.011553	2.558468
HIST1H1A	-1.03222	5.249701	-5.77463	0.000451	0.03452	0.381872
SCNN1A	-1.03907	7.479328	-8.90881	2.28E-05	0.007694	3.314433
HIST1H2AB	-1.07344	8.065709	-5.58109	0.000562	0.038202	0.158669
GLP2R	-1.11017	7.780635	-11.02	4.82E-06	0.004101	4.731521
MYPN	-1.12719	7.42583	-10.1703	8.70E-06	0.005247	4.203973
HIST1H2BI	-1.13604	6.08771	-5.2416	0.000836	0.046549	-0.24654
CCL2	-1.14721	5.314616	-12.6518	1.72E-06	0.002378	5.610076
S100A4	-1.15053	6.164204	-10.5789	6.51E-06	0.004688	4.464307
KIF20A	-1.15804	8.626588	-11.5903	3.31E-06	0.003538	5.057388
HSPA1A	-1.18133	9.35104	-11.0628	4.68E-06	0.004075	4.756747
ZNF831	-1.18556	5.812955	-6.95585	0.00013	0.01809	1.628511
HIST1H3A	-1.18869	6.441841	-7.55158	7.37E-05	0.013674	2.1881
HSPA1B	-1.20391	9.316029	-11.1242	4.49E-06	0.004075	4.792689
GATSL2	-1.22061	7.183912	-9.40487	1.54E-05	0.006705	3.680643
METTL7A	-1.241	6.802659	-9.19234	1.82E-05	0.006822	3.526483
NPY4R	-1.3319	6.836449	-8.96261	2.18E-05	0.007407	3.355245
LXN	-1.34896	6.031784	-6.28611	0.000258	0.025948	0.945372
GATSL1	-1.37709	7.131902	-10.0593	9.43E-06	0.005498	4.131046
ALDH1A3	-1.40489	7.11515	-11.6537	3.18E-06	0.003504	5.092274
OLFML1	-1.6252	7.527223	-8.44414	3.35E-05	0.00908	2.950342

**Table 2:** Pharmacological and molecular properties of baicalein.

MW	AlogP	Hdon	Hacc	OB (%)	Caco-2	BBB	DL	FASA-	TPSA	RBN	HL
270.25	2.33	3	5	33.52	0.63	-0.05	0.21	0.36	90.9	1	16.25

**Table 3:** Predicted targets of baicalein.

Protein name	Gene symbol
Prostaglandin G/H synthase 1	PTGS1
Androgen receptor	AR
Prostaglandin G/H synthase 2	PTGS2
Heat shock protein HSP 90	HSP90AA1
Heat shock protein HSP 90	HSP90AB1
mRNA of PKA Catalytic Subunit C-alpha	PRKACA
Dipeptidyl peptidase IV	DPP4
Phosphatidylinositol-4,5-bisphosphate 3-kinase catalytic subunit, gamma isoform	PIK3CG
CGMP-inhibited 3′,5′-cyclic phosphodiesterase A	PDE3A
Trypsin-1	PRSS1
Nuclear receptor coactivator 2	NCOA2
Nuclear receptor coactivator 1	NCOA1
Calmodulin	CALM1
Transcription factor p65	RELA
RAC-alpha serine/threonine-protein kinase	AKT1
Vascular endothelial growth factor A	VEGFA
Apoptosis regulator Bcl-2	BCL2
Proto-oncogene c-Fos	FOS
Apoptosis regulator BAX	BAX
Matrix metalloproteinase-9	MMP9
Caspase-3	CASP3
Cellular tumor antigen p53	TP53
Hypoxia-inducible factor 1-alpha	HIF1A
Fos-related antigen 1	FOSL1
Fos-related antigen 2	FOSL2
Cell division control protein 2 homolog	CDK1
G2/mitotic-specific cyclin-B1	CCNB1
Myeloperoxidase	MPO
Aryl hydrocarbon receptor	AHR
Insulin-like growth factor II	IGF2
Cytochrome c	CYCS
Arachidonate 12-lipoxygenase, 12S-type	ALOX12
Nuclear factor of activated T-cells, cytoplasmic 1	NFATC1
Tudor domain-containing protein 7	TDRD7
Egl nine homolog 1	EGLN1
NADPH oxidase 5	NOX5
Fatty acid-binding protein, epidermal	FABP5
Apolipoprotein D	APOD

## Data Availability

The data used to support the findings of this study are available from the corresponding author upon request.
